# Mycobacterial infection induces a specific human innate immune response

**DOI:** 10.1038/srep16882

**Published:** 2015-11-20

**Authors:** John D. Blischak, Ludovic Tailleux, Amy Mitrano, Luis B. Barreiro, Yoav Gilad

**Affiliations:** 1 Department of Human Genetics, University of Chicago, Chicago, Illinois, USA; 2 Committee on Genetics, Genomics, and Systems Biology, University of Chicago, Chicago, Illinois, USA; 3 Mycobacterial Genetics Unit, Institut Pasteur, Paris, France; 4 Department of Genetics, CHU Sainte-Justine Research Center, Montreal, Québec, Canada; 5 Department of Pediatrics, University of Montreal, Montreal, Québec, Canada

## Abstract

The innate immune system provides the first response to infection and is now recognized to be partially pathogen-specific. *Mycobacterium tuberculosis* (MTB) is able to subvert the innate immune response and survive inside macrophages. Curiously, only 5–10% of otherwise healthy individuals infected with MTB develop active tuberculosis (TB). We do not yet understand the genetic basis underlying this individual-specific susceptibility. Moreover, we still do not know which properties of the innate immune response are specific to MTB infection. To identify immune responses that are specific to MTB, we infected macrophages with eight different bacteria, including different MTB strains and related mycobacteria, and studied their transcriptional response. We identified a novel subset of genes whose regulation was affected specifically by infection with mycobacteria. This subset includes genes involved in phagosome maturation, superoxide production, response to vitamin D, macrophage chemotaxis, and sialic acid synthesis. We suggest that genetic variants that affect the function or regulation of these genes should be considered candidate loci for explaining TB susceptibility.

The innate immune system provides the first line of defense against microbial pathogens. Broadly speaking, innate immune cells recognize foreign molecules through pattern recognition receptors (PRRs), e.g. Toll-like receptors (TLRs), which bind to highly-conserved pathogenic motifs known as pathogen-associated molecular patterns (PAMPs)^1^,^2^. In addition, innate immune cells recognize damage-associated molecular patterns (DAMPs) of host molecules released by infected cells^3^. The initial innate response involves the release of proinflammatory cytokines and lipids to recruit and activate other immune cells, phagocytosis of the pathogen, and apoptosis^4^. If the infection persists, the phagocytes stimulate the adaptive immune system by presenting antigens to activate T and B cells. In contrast to the highly specific adaptive immune response, the innate immune response has traditionally been viewed as a general response to infection.

Yet, more recent work revealed that the innate immune system also produces a pathogen-specific response in addition to the general response^5^,^6^,^7^,^8^. Furthermore, this pathogen-specific innate response can in turn affect the specificity of the adaptive immune response by directing the differentiation of T cells into distinct subtypes^9^. That said, though we developed an appreciation for the importance of the specific innate immune response, we still do not know the extent to which the innate immune response differs between infections nor fully understand the consequences of specific innate immune responses for fighting pathogens. One of the first challenges is to distinguish the unique immune response to a specific pathogen from the large core more general response.

The pathogen-specific innate immune response is determined, at least in part, by the specificity of the PRRs of the host immune cell. Each PRR binds to its specific targets and activates certain downstream signaling pathways^10^. For example, treatment of mouse dendritic cells with lipopolysaccharide (LPS), which is found on the outer membrane of gram-negative bacteria, or with PAM3CSK4 (PAM), which is a synthetic lipoprotein that mimics those found on both gram-negative and gram-positive bacteria, induce different transcriptional response programs, because the two antigens are bound by TLR4 and TLR2, respectively^11^. Different pathogens not only stimulate different PRRs, but they have also evolved different evasion mechanisms to manipulate the innate immune response^2^,^12^,^13^,^14^. These evasion strategies likely also contribute to the specificity of the response to different pathogens.

In the context of evasion strategies, the case of *Mycobacterium tuberculosis* (MTB), the causative agent of tuberculosis (TB), is especially interesting. In order to increase its success inside alveolar macrophages - the primary cells that target MTB upon infection - MTB subverts the immune response through various mechanisms. MTB disrupts phagosomal maturation, thus preventing acidification by vesicular proton pumps and lysosomal fusion^12^,^15^,^16^, and delays stimulation of the adaptive immune system by inducing host expression of anti-inflammatory cytokines^17^,^18^. In order to achieve these manipulations, MTB must be able to secrete bacterial effectors from the phagosome into the cystosol where they can interact with host factors^19^. For this reason, the ESX-1 secretion system of MTB is critical for virulence because it permeabilizes the phagosome membrane^20^,^21^. Not only does this membrane permeabilization provide a means for bacterial molecules to access the cytosol, but at later timepoints MTB has been observed to have escaped into the cytosol^19^. One well-studied consequence of phagosomal permeability is the detection of MTB DNA in the cytosol by the host sensor cGAS (*MB21D1*) and subsequent activation of the STING (*TMEM173*) pathway^22^,^23^,^24^,^25^. These signaling events result in immune responses that are both beneficial and detrimental to MTB survival. On the one hand, the expression of anti-viral type I interferons are increased, a response which has been observed to benefit the growth of MTB and other bacteria^26^. On the other hand, MTB is targeted for destruction via autophagy, a key defense mechanism for fighting intracellular pathogens^27^. Thus the survival or destruction of MTB inside the macrophage depends on complex interactions between secreted bacterial effectors and host immune factors.

While the adaptive immune system is needed to prevent the spread of MTB and subsequent onset of TB, infected individuals do not become immunized against future MTB infections. This property may be related to the difficulty to develop an effective vaccine for adult TB (the current vaccine, bacillus Calmette–Guérin, BCG, is partly effective in children, much less so in adults)^28^.

Interestingly from a human genetics viewpoint, there are large inter-individual differences in susceptibility to developing TB. While it is estimated that roughly a third of the human population is latently infected with MTB, only approximately 10% of healthy infected individuals will develop active TB (immunocompromised individuals, e.g. HIV-infected, develop active TB at a much higher frequency)^29^. Despite an inference for a strong individual genetic component to TB susceptibility, the genetic architecture remains largely unknown^30^,^31^,^32^,^33^. There have been quite a few reports of candidate-gene associations, but genome wide scans have only identified two weak associations with disease susceptibility^34^,^35^,^36^.

To begin addressing this gap, we have previously investigated genetic variation that is associated with inter-individual differences in the transcriptional response of human phagocytes to infection with MTB^37^. We found 102 and 96 genes that were associated with an expression QTL (eQTL) only pre- or post-infection, respectively. We refer to these loci as response eQTLs since their association with gene expression is affected by MTB infection. Interestingly, these response eQTLs were enriched for significant signal in a genome wide association study of TB susceptibility^34^. However, it is unknown if the genes associated with these response eQTLs are induced specifically in response to infection with MTB or are a part of the core innate immune response.

In order to characterize the innate immune response specific to MTB infection and better understand the role of the response eQTL-associated genes in the innate immune response, we infected macrophages isolated from a panel of six healthy individuals with a variety of bacteria. In addition to MTB, we chose both related mycobacteria and more distantly related bacteria.

## Results

### Bacterial infection induces large changes in gene expression

To learn about the immune response to infection with different bacteria, with a particular emphasis on *Mycobacterium tuberculosis* (MTB), we investigated the *in vitro* gene regulatory response of macrophages to infection with multiple MTB stains, related mycobacterial species, and other bacterial species (Table 1). Specifically, we infected cultured macrophages with either MTB H37Rv, which is a common strain often used in laboratory experiments^38^; MTB GC1237, which is a strain of the highly virulent Beijing family^39^; bacillus Calmette-Guérin (BCG), which is attenuated *Mycobacterium bovis* used for vaccinations; *Mycobacterium smegmatis*, which is non-pathogenic; or heat-killed MTB H37Rv. In order to compare the response to infection with mycobacteria to the response to infection with other bacteria, we also included infection treatments with *Yersinia pseudotuberculosis* (gram-negative), *Salmonella typhimurium* (gram-negative), or *Staphylococcus epidermidis* (gram-positive).

**Figure 1:**
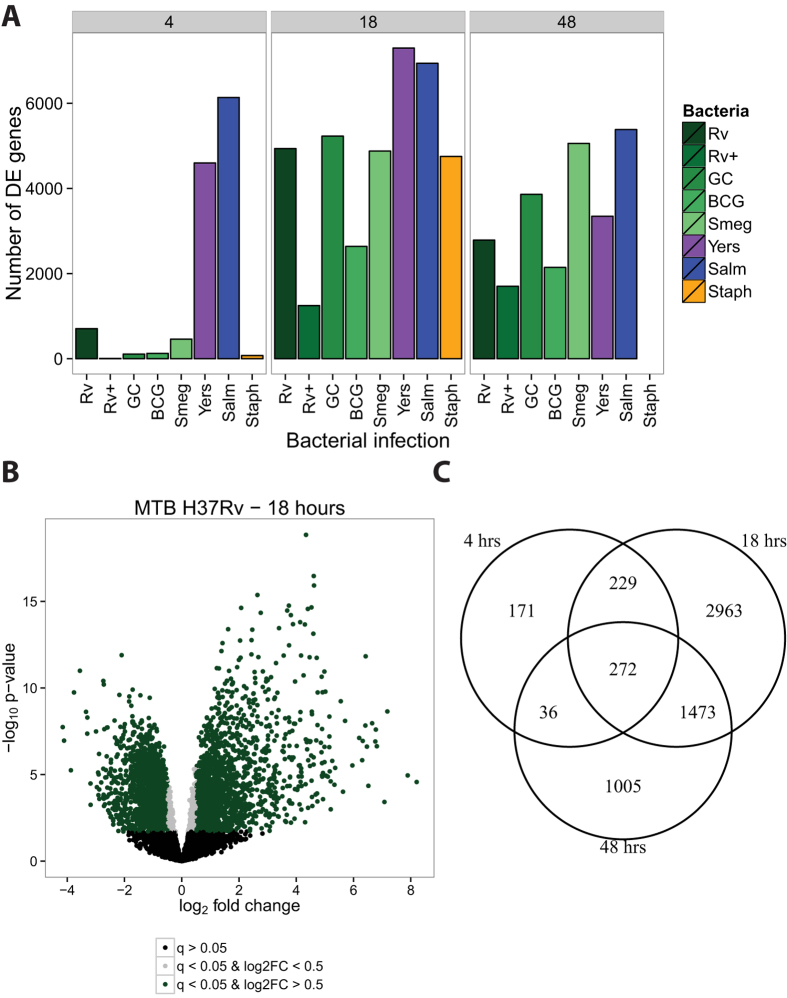
Differential expression analysis. We tested for differentially expressed genes for each bacterial infection by comparing it to its time-matched control. (**A**) We classified genes with q-value < 5% as differentially expressed. The mycobacteria are labeled in shades of green. (**B**) As expected, there were large transcriptional changes 18 hours post-infection with MTB H37Rv. Genes with q-value < 5% and absolute log_2_ fold change greater than 0.5 are labeled green, those with q-value < 5% and absolute log 2 fold change less than 0.5 are labeled grey, and non-differentially expressed genes are labeled black. (**C**) The overlap in differentially expressed genes identified at 4, 18, and 48 hours post-infection with MTB H37Rv.

We infected monocyte-derived macrophages from six individuals with the bacteria described above (including a non-infected control) and extracted RNA at 4, 18, and 48 hours post-infection (see Methods; Supplementary Fig. S1). We assessed RNA quality using the Agilent Bioanlyzer (Supplementary Table S6) and sequenced the RNA to estimate gene expression levels. Detailed descriptions of our data processing, quality control analyses, and statistical modeling are available in the Methods section. Briefly, we mapped the short RNA-seq reads to the human genome (hg19) using the Subread algorithm^40^, discarded reads that mapped non-uniquely, and counted the number of reads mapped to each protein-coding gene. We normalized the read counts using the weighted trimmed mean of M-values algorithm (TMM)^41^, corrected for confounding "batch" effects (Supplementary Fig. S2), and used limma + voom^42^,^43^,^44^ to test for differential expression (DE) between cultures infected with each bacteria and their time-matched controls (Supplementary Table S2). Using this approach we initially observed the following general patterns: at four hours post-infection, only *Y. pseudotuberculosis* and *S. typhimurium* elicited a strong transcriptional response (Fig. 1A); at 18 hours post-infection, all the bacteria had elicited a strong immune response (Fig. 1A,B); and at 48 hours post-infection, all the bacteria continued stimulating the immune response (Fig. 1A), however, many of the DE genes were not shared between the 18 and 48 hour timepoints (Fig. 1C). Of note, at 48 hours post-infection we were unable to collect RNA from macrophages infected with *S. epidermidis* (see Methods).

### Joint analysis identifies bacteria-specific response genes

In order to learn about variation in the innate immune response to bacterial infection, we identified genes whose regulation was altered by treatment with specific bacteria at specific timepoints. We first used a naïve approach whereby we determined all the pairwise overlaps between lists of DE genes across treatments (Supplementary Table S7). The caveat of this strategy is that incomplete power can result in overestimating the difference between treatments. In order to account for incomplete power to detect DE genes when performing multiple pairwise comparisons, we performed a joint Bayesian analysis, which we implemented using the R/Bioconductor package Cormotif^45^ (see Methods for more details). Using this approach, we classified genes into regulatory patterns based on their expression levels following each of the bacterial infections.

**Figure 2:**
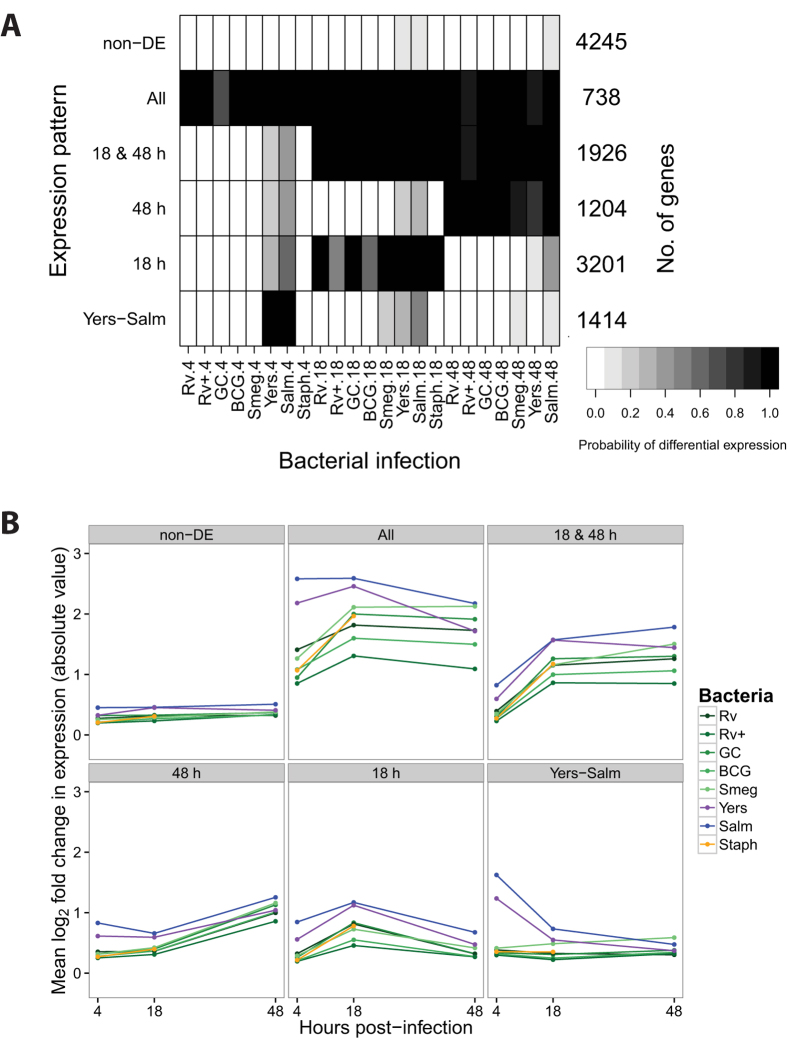
Joint Bayesian analysis. (**A**) Joint analysis of gene expression data from all three timepoints with Cormotif^45^ identified six expression patterns: "non-DE", "Yers-Salm", "18 h", "48 h", "18 & 48 h", and "All". The shading of each box represents the posterior probability that a gene assigned to the expression pattern (row) is differentially expressed in response to infection with a particular bacteria (column), with black representing a high posterior probability and white a low posterior probability. (**B**) Each data point is the mean log_2_ fold change in expression (absolute value) in response to infection with the given bacteria for all the genes assigned to the particular expression pattern.

First, we examined the data across all the bacteria-time combinations. Initially we built a model that classified genes into one of 14 separate patterns based on their expression levels after each infection relative to their expression level in the non-infected control (Supplementary Fig. S3). However, we found that a model with only six expression patterns (Fig. 2; Supplementary Tables S3,S4), where a subset of the original 14 patterns are combined, is more intuitive from a biological perspective; thus we proceeded with the reduced model. Broadly speaking, we classified genes as responding in the early, middle, or late stages of infection, and we characterized the response as temporary or sustained. Pattern "non-DE" includes 4,245 genes whose expression levels were unchanged in all the experiments. Pattern "Yers-Salm" includes 1,414 early response genes whose expression levels changed at four hours post-infection with either *Y. pseudotuberculosis* or *S. typhimurium*, but not after infection with other bacteria. The genes in this pattern are enriched for gene ontology (GO) annotations related to type I interferon signaling (e.g. *SP100*, *IFI35*, *STAT2*), antigen presentation (*HLA-A*, *PSME1*, *CTSS*), and apoptosis (*CASP8*, *TRADD*, *FADD*) (Supplementary Table S5). Pattern "18 h" includes 3,201 middle response genes whose expression levels changed exclusively at 18 hours post-infection in response to all bacteria and is enriched for GO annotations related to apoptosis (e.g. *E2F1*, *TP53*, *WWOX*). Pattern "48 h" includes 1,204 late response genes whose expression levels changed at 48 hours and is enriched for GO annotations related to phagocytosis (e.g. *MFGE8*, *COLEC12*) and tumor necrosis factor-mediated signaling (e.g. *STAT1*, *TRAF2*, *TNFRSF14*). Pattern "18 & 48 h" includes 1,926 middle-sustained response genes whose expression levels changed at 18 and 48 hours and is enriched for GO annotations related to the regulation of phagocytosis (e.g. *CD36*) and TLR signaling (*TLR1*, *TLR2*, *MYD88*). Lastly, pattern "All" includes 738 early-sustained genes whose expression levels changed after infection with all the bacteria across all three timepoints and is enriched for GO annotations related to type I interferon signaling (e.g. *IRF1*, *SOCS1*, *IFIT3*), cytokine secretion (*TNF*, *IL10*, *LILRB1*), and apoptosis (e.g. *IRF7*, *BCL2A1*, *MCL1*).

**Figure 3:**
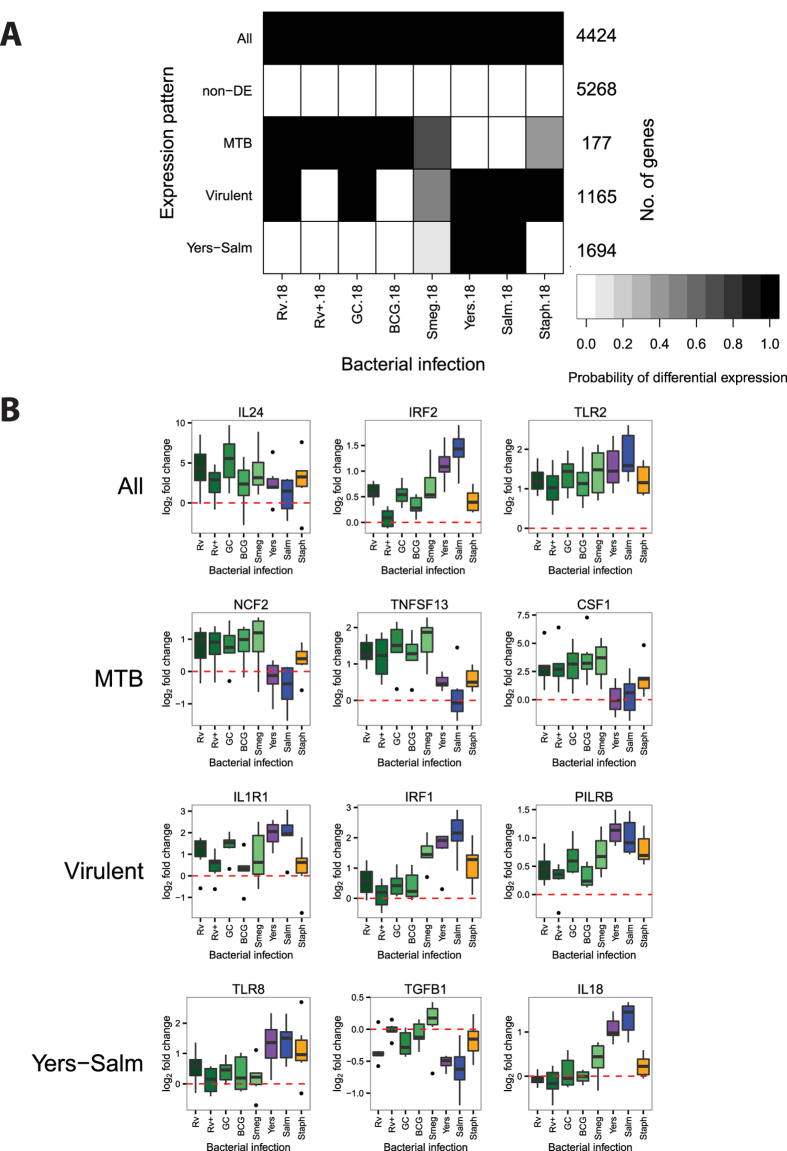
Joint Bayesian analysis - 18 hours post-infection. (**A**) Joint analysis of gene expression data from 18 hours post-infection with Cormotif identified five expression patterns: "Yers-Salm", "Virulent", "MTB", "non-DE", and "All". (**B**) Example genes from the different expression patterns.

Next, we tested for more specific patterns by performing Cormotif separately on the data from the middle (18 h) and late (48 h) stages of infection. At 18 hours post-infection, we identified five separate expression patterns (Fig. 3; Supplementary Tables S3,S4). Pattern "non-DE" includes 5,268 genes whose expression levels were unchanged across all infections. Pattern "All" includes 4,424 genes whose expression levels were affected by all infections (e.g. *IL24*, *IRF2*, *TLR2*). Pattern "MTB" includes 177 genes whose expression levels changed specifically in response to infection with mycobacteria (e.g. *NCF2*, *TNFSF13*, *CSF1*). These genes had a high posterior probability of being DE 18 hours after infection with MTB H37Rv, heat-killed H37Rv, MTB GC1237, and BCG. Furthermore, the gray shading for *M. Smegmatis* (Fig. 3A) signified an intermediate posterior probability for DE. In essence, this pattern is a merger of two sets of genes that were not large enough to be separated: one set that was DE across all five mycobacteria and another that was only DE after infection with the MTB strains and the closely-related BCG, but not *M. Smegmatis*. Pattern "Virulent", in contrast, includes 1,165 genes whose expression levels were less strongly changed after infection with heat-inactivated MTB H37Rv or the attenuated vaccine strain BCG compared to the other bacteria (e.g. *IL1R1*, *IRF1*, *PILRB*). Also the genes in this category only have an intermediate probability of responding to the non-pathogenic *M. smegmatis*. Lastly, pattern "Yers-Salm" includes 1,694 genes whose expression levels changed preferentially after infection with *Y. pseudotuberculosis* or *S. typhimurium* (e.g. *TLR8*, *TGFB1*, *IL18*).

**Figure 4:**
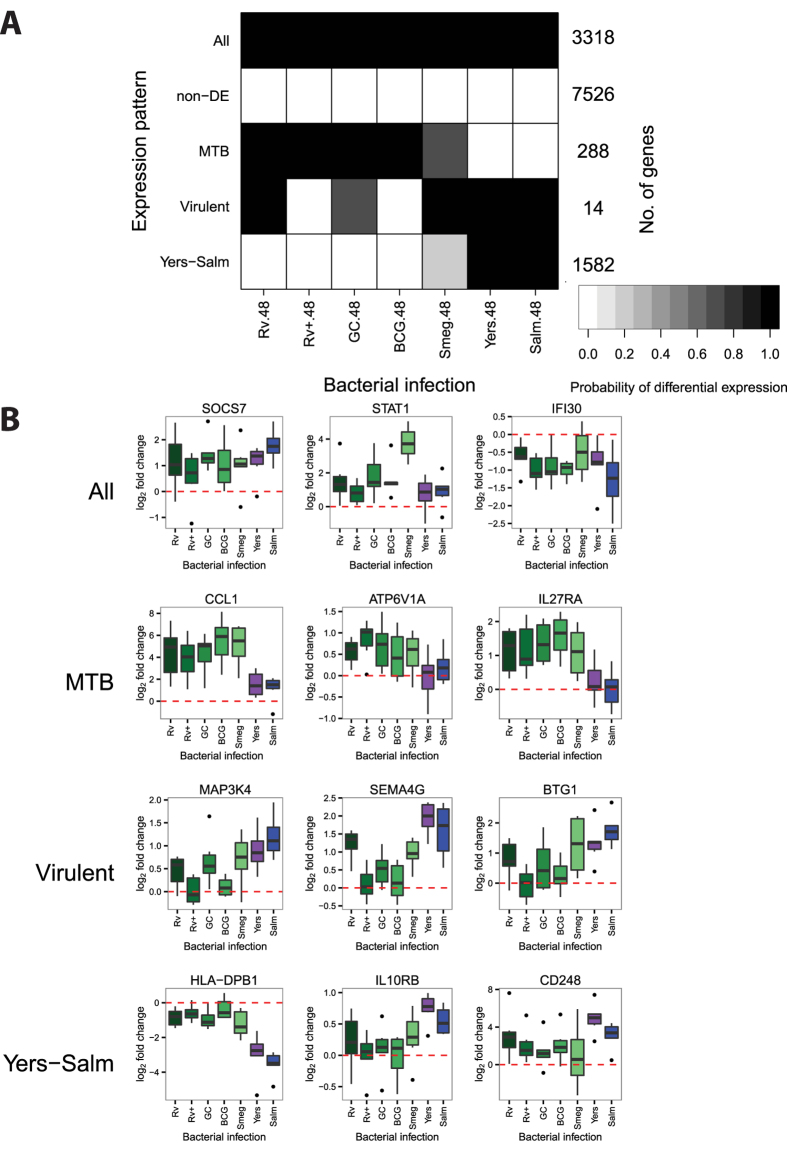
Joint Bayesian analysis - 48 hours post-infection. (**A**) Joint analysis of gene expression data from 48 hours post-infection with Cormotif identified five expression patterns: "Yers-Salm", "Virulent", "MTB", "non-DE", and "All". (**B**) Example genes from the different expression patterns.

At 48 hours post-infection, we also discovered five expression patterns (Fig. 4; Supplementary Tables S3,S4). While many of the patterns have similar specificities to those observed at 18 hours post-infection, there is only little overlap across timepoints with respect to the genes comprising the patterns. For example, pattern "Yers-Salm" at 48 hours includes 1,582 genes whose expression levels changed strongly after infection with *Y. pseudotuberculosis* or *S. typhimurium* (e.g. *HLA-DPB1*, *IL10RB*, *CD248*), but only 263 of these genes are also in the corresponding pattern when we considered the data from the 18 hour timepoint. Similarly, at the 48 hour timepoint, pattern "MTB" includes 288 genes whose expression levels changed preferentially after infection with mycobacteria (e.g. *CCL1*, *ATP6V1A*, *IL27RA*), but only 33 of these genes are in the corresponding pattern at the 18 hour timepoint. Pattern "Virulent" includes 14 genes whose expression levels were not changed after infection with heat-inactivated MTB H37Rv or the attenuated vaccine strain BCG (e.g. *MAP3K4*, *SEMA4G*, *BTG1*), and only one of these also belongs to the pattern "Virulent" at 18 hours post-infection.

### Infection-induced response eQTLs are shared across bacterial infections

**Figure 5:**
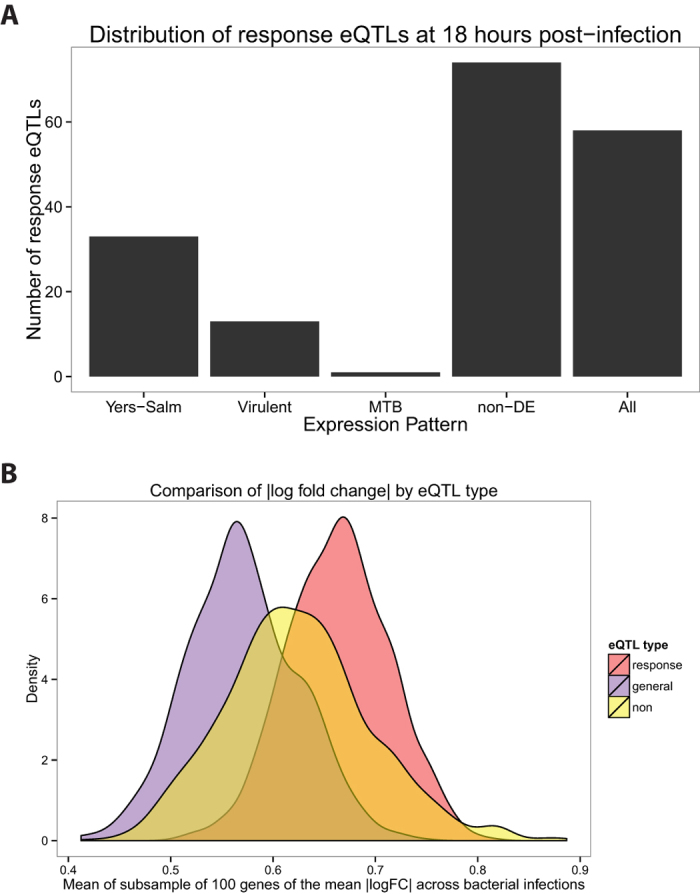
Response eQTLs at 18 hours post-infection. (**A**) We counted the number of response eQTLs from Barreiro *et al.*^37^ (179 out of the 198 were also expressed in our study) in each of the five gene expression patterns at 18 hours post-infection (Fig. 3). (**B**) We compared the mean log_2_ fold change in expression across the 8 bacterial infections at the 18 hour timepoint for three classes of genes: response eQTL genes (red), general eQTL genes (purple), and non-eQTL genes (yellow) (see Methods for details).

Using the gene expression patterns we identified by applying the joint analysis approach, we investigated the specificity of previously identified response eQTLs to infection with MTB H37Rv^37^. Since the response eQTLs were identified at 18 hours post-infection, we investigated the distribution of genes associated with response eQTLs among the five patterns we found at that timepoint (Fig. 5A). Only one gene associated with a response eQTL was also DE specifically in response to MTB (*CMAS*). Otherwise, most of the response eQTL-associated genes were classified as either DE following infection with all bacteria or not DE in any infection. That a large proportion of the genes associated with response eQTLs were not DE in any of these experiments is likely due to the fact that the eQTL study was performed in dendritic cells whereas our data were collected from macrophages. Overall, our observations suggest that most of the previously identified response eQTLs are genetic variants that affect the human innate immune response to bacterial infection in general, and not specifically the response to MTB H37Rv.

To provide further broad support for the interpretation that the response eQTL genes are important for the innate response to bacterial infection in general, we considered the log_2_ fold change in expression values following infection (Fig. 1). For each gene, we calculated the mean log_2_ fold change in expression level across the eight bacterial infections at the 18 hour timepoint. Next, we compared the absolute values of the log_2_ fold change in expression between genes associated with response eQTLs, genes associated with general eQTLs (i.e. genes associated with an eQTL pre- and post-infection), and genes not found to be associated with an eQTL. Since there was a large difference in the number of genes in these three classes, we subsampled genes from each and calculated the mean of the absolute values (and repeated this process 1000 times). We found that the expression level of genes associated with response eQTLs is altered to a larger degree (significantly higher effect size; *P* < 2.2 × e^−16^; Fig. 5B) following infection compared to the genes in the other two classes.

## Discussion

### Bayesian analysis identified mycobacteria-specific response genes

In order to identify general and treatment-specific gene regulatory responses, we performed a joint Bayesian analysis of the data using Cormotif^45^. By jointly analyzing the data, as opposed to comparing overlaps between independent lists of differentially expressed genes generated using an arbitrary cutoff, we minimized the identification of specific responses due to false negatives (i.e. genes that appear to be differentially expressed in response to a subset of bacterial infections when in reality the response is similar across all the infections). Similar to previous observations^5^,^6^, we found a large core transcriptional response to infection. However, we also identified a novel subset of genes whose regulation is preferentially altered in response to infection with mycobacteria but not to the other bacteria we tested. Since these responses are unique to infection with mycobacteria (at least in the context of our study design), they may be promising candidates for future studies that focus on the mechanisms by which mycobacteria successfully subvert the human innate immune response. Since this study does not extend to investigation of mechanisms, we do not have empirical data with which to prioritize such possible candidate genes. Yet, the reported functions of many of these genes often suggest mechanisms that are relevant, and often quite specific, to MTB infection. Prioritizing candidate genes in this way is not statistically valid, and one can argue (and indeed, this has been shown^46^) that any list of genes can be scrutinized to yield "interesting relevant stories". We therefore offer these details in the context of a discussion (rather than "results"), to provide one set of alternative explanations for our findings, and generate ideas for further investigations.

For example, when we focused on the mycobacteria-specific regulatory response 18 hours post-infection, we noticed an intriguing number of genes that are involved in phagosome maturation (Supplementary Fig. S4). Broadly speaking, phagocytosed bacteria are killed by vesicular proton pumps, which lower the pH inside the phagosome, and lysosomal fusion. This process occurs once a phagosome has matured through a series of steps mediated by the exchange of Rab GTPases^47^,^48^. A unique property of mycobacteria is their ability to survive inside the macrophage by inhibiting phagosome maturation^16^. As part of this strategy, the bacterium recruits *RAB22A* to MTB-containing phagosomes^49^. Indeed, we found the *RAB22A* gene to be upregulated in response to infection with mycobacteria (Supplementary Table S4). Similar GTPases whose regulation was altered following mycobacterial infections include *RAP2A* (upregulated), *RAB3A* and *RAB33A* (both downregulated). In addition, the vesicular (v)-ATPase subunit *ATP6V1D* was exclusively upregulated in response to mycobacterial infection. Thus, the mycobacteria-specific response we identified includes genes putatively involved in mycobacteria-specific survival mechanisms.

An additional intriguing example involves the *NCF2* gene. This is a potential candidate gene whose expression level was affected specifically by infection with mycobacteria at 18 hours post-infection. Neutrophil cytosolic factor 2 (*NCF2*, also known as *p67phox*) is a subunit of the phagocyte NAPDH oxidase, which is responsible for generating reactive oxygen species used to fight intracellular pathogens^50^,^51^,^52^,^53^,^54^,^55^. These reactive oxygen species may also serve a signaling role in activating other immune cell types to ensure proper granuloma formation and killing of mycobacteria^56^. Loss-of-function mutations in subunits of the NAPDH oxidase cause chronic granulomatous disease (CGD)^55^, which is characterized by the formation of granulomas throughout the body due to the inability of phagocytes to kill the ingested pathogens. In contrast to wild type animals, mice with mutations in subunits of the phagocyte NAPDH oxidase develop tuberculosis after infection with the vaccine strain, BCG^56^. Humans who are administered the vaccine before being diagnosed with CGD also develop the disease^55^.

At 48 hours post-infection (Fig. 4), the mycobacteria-specific response was enriched with genes annotated (based on GO) as having a role in "response to vitamin D" (Supplementary Table S5; Supplementary Fig. S5). Individuals with low circulating levels of vitamin D are more susceptible to developing tuberculosis^57^,^58^, and vitamin D has been investigated as a supplemental therapy for the treatment of tuberculosis, though with mixed results^59^,^60^,^61^,^62^. Vitamin D has been found to be important for innate immune cells to fight MTB^63^,^64^,^65^; however, it is also an important pathway for generic bactericidal activity^66^. Consistent with its role in the innate immune response, both the enzyme that converts vitamin D to its active form (*CYP27B1*) and its receptor (*VDR*) are upregulated in response to any of the infections (pattern "All"; Fig. 4). Yet, the regulation of other genes involved in the response to vitamin D was only affected by infection with MTB. *PIM1*, a serine/threonine kinase that binds the VDR and enhances transcription of its target genes^67^, is upregulated in response to the mycobacteria (pattern "MTB", Fig. 4). Interestingly, the increased expression level of *PIM1* in T-cells was successfully used in a six-gene classifier of patients with active versus latent TB infections^68^. Another gene, the chemokine *CXCL10* (also known as interferon gamma-induced protein 10 or IP-10), is also upregulated in response to mycobacterial infection (pattern "MTB", Fig. 4). Discordant with the observed increase in expression of *CYP27B1*, *VDR*, and *PIM1* in response to infection, treatment with vitamin D usually leads to the reduction of *CXCL10* expression and secretion in multiple cell types^69^,^70^,^71^. In fact, supplementation with vitamin D decreased serum levels of CXCL10 in TB patients^72^. This suggests that the immunosuppresive effect of vitamin D signaling is insufficient to overcome the pro-inflammatory response to mycobacterial infection. This observation is in concordance with past studies which found increased expression of *CXCL10*, as well as increased secretion level from macrophages, following infection with MTB^64^,^73^. Interestingly, a polymorphism in *CXCL10* was found to be associated with susceptibility to tuberculosis in a Chinese population^74^,^75^. Overall, these observations provide support for the importance of vitamin D signaling for specifically fighting mycobacterial infections.

Another gene of interest from the mycobacteria-specific expression pattern at 48 hours post-infection is chemokine (C-C motif) ligand 1 (*CCL1*), which stimulates migration of human monocytes^76^ (Fig. 4B). Thuong *et al.* identified *CCL1* as being induced to a greater extent in MTB-infected macrophages (4 hours post-infection) isolated from individuals with pulmonary TB compared to macrophages from individuals with latent TB infections^77^. Put together, our observations and those of Thuong and colleagues suggest that *CCL1* is involved in the pathogenesis of TB. Further supporting this notion, Thuong *et al.* also found a genetic association between variants in the *CCL1* region and TB susceptibility^77^. However to date, subsequent genetic association studies investigating *CCL1* have reported mixed results^78^,^79^.

One caveat of the joint Bayesian analysis is that we were not able to classify genes into unusual patterns (because this approach can only discover expression patterns shared by a large number of genes and, by definition, only few genes fall into "unusual" patterns). For example, unusual patterns of interest include changes in expression specifically in response to some but not all of the mycobacterial infections. One gene that satisfied this pattern is the dual specificity phosphatase 14 (*DUSP14*). We specifically examined the expression data for this gene because it was previously associated with an MTB infection response eQTL in dendritic cells^37^, and consequently when the eQTL results were used as a prior - *DUSP14* was found to be significantly associated with TB susceptibility. Moreover, knocking down *DUSP14* expression via siRNA in murine macrophages resulted in a lower bacterial load 90 hours post-infection with MTB H37Rv^80^. In our joint Bayesian analysis, *DUSP14* was not classified as one of the genes whose regulation was altered in response to infection with mycobacteria. Yet, *DUSP14* was upregulated at 18 hours post-infection with MTB H37Rv (q-value: 16%), MTB GC1237 (q-value: 3%), and BCG (q-value: 9%); and downregulated post-infection with *S. typhimurium* (q-value: 9%) (Supplementary Fig. S6). Thus, our data lends further support for the role of *DUSP14* as a TB susceptibility gene.

### Little evidence for strain-specific transcriptional response to infection

There are six major families of MTB that differ in their geographic distribution and virulence^81^,^82^. Strains from these families are known to differ in their growth rates inside macrophages^83^, expression levels of bacterial genes^84^,^85^, and cell wall lipid composition^86^. Previous studies have found that different MTB strains induce different innate immune responses in human cell lines and other infection models^87^. A dominate narrative is that MTB strains from East Asia, referred to as the Beijing family (Gagneux *et al.* classified it as MTB lineage 2^81^), are more virulent because they induce a lower proinflammatory immune response compared to the common laboratory strains^88^,^89^,^90^,^91^,^92^. However, other studies have reported the opposite, namely that Beijing strains induce a larger proinflammatory response^93^, or a conflicting response in which various pro- and anti-inflammatory cytokines are differentially regulated^94^,^95^ compared to laboratory strains.

In our study, albeit with a small sample size, we found no marked differences between the transcriptional response to infection with MTB H37Rv or MTB GC1237, a Beijing strain (Supplementary Fig. S7; Supplementary Table S8). Furthermore, the pro-inflammatory cytokines *TNF* and *IL6* and the anti-inflammatory cytokine *IL10* were strongly upregulated in response to both strains of MTB (Supplementary Fig. S8). This observation is in concordance with Wu *et al.*, who also reported no apparent difference in the transcriptional response of THP-1 cells to infection with MTB H37Rv versus multiple Beijing strains^96^. Thus the increased virulence of the Beijing family of MTB strains may be due to mechanisms not assayed in this study such as post-transcriptional effects, cell-cell signaling, and environmental stimuli. It should be noted, however, that not all Beijing strains are equally virulent^97^,^98^ and that MTB H37Rv is a laboratory-adapted strain that has evolved independently in different laboratories^99^.

### Differences in response to virulent versus attenuated pathogens are not mycobacteria-specific

To better understand the interaction between MTB and macrophages, we included in our study both virulent mycobacteria (MTB strains H37Rv and GC1237) and attenuated mycobacteria (heat-inactivated MTB H37Rv and the vaccine strain BCG). Overall, the response to infection with either virulent or attenuated mycobacteria was similar (Figs 3 and 4). This observation was unsurprising because it has been previously demonstrated that infections with inactivated pathogens (in fact, even individual pathogen components) are able to largely recapitulate the transcriptional response to infection^5^,^6^,^7^,^8^. In other words, as expected, the transcriptional response to infection is largely driven by the antigens present.

Yet, the responses to inactivated pathogens or individual pathogen components in past studies were not identical to the responses to live pathogens, suggesting a potential role for bacterial manipulation of the immune response. For example, it is known that BCG lacks the locus containing the ESX-1 secretion system, which is critical for MTB virulence^100^,^101^,^102^,^103^. In our study we also observed differences between the response to virulent and attenuated mycobacteria. Specifically, there are 1,165 genes in the expression pattern "Virulent" at 18 hours post-infection (Fig. 3) and 14 genes that comprise of the “Virulent” pattern at 48 hours post-infection (Fig. 4). Importantly, these genes are also differentially expressed in response to the other virulent infections in our study, and thus they are not specifically due to the manipulations of the host cell by virulent mycobacteria.

We attempted to identify a gene expression pattern that specifically represented differences in virulence only in the mycobacteria, yet we never saw such a pattern. It is important to note that had we simply performed a simple pairwise analysis of the overlap of DE genes between MTB and BCG infections, our results would be quite different (Supplementary Table S7). Yet, a pairwise analysis is misleading in the context of the entire study. Indeed, by accounting for incomplete power by using the joint Bayesian model and including other bacterial species, we avoided attributing many differentially expressed genes specifically to the differences in the immune evasion mechanisms used by MTB and BCG. We conclude that either a larger sample size or a different experimental system is required to find specific differences between the response to infection with MTB and BCG.

### Previously identified response eQTLs affect response to bacterial infection in general

In a previous study, we identified response eQTLs that were associated with gene expression levels in MTB-infected human dendritic cells. We investigated the expression pattern of genes associated with the response eQTLs in our study. Using the five expression patterns identified by the joint Bayesian analysis at 18 hours post-infection, we examined the distribution of response eQTL genes and discovered that these genes were not enriched in the mycobacteria-specific expression pattern (Fig. 5A). Instead, many were differentially expressed across all the infections (pattern "All"). Thus, response eQTLs modulate the inter-individual response to infection with diverse types of bacteria. That said, one gene was both associated with a response eQTL and specifically differentially expressed following mycobacterial infection. Though this result does not represent a significant enrichment of response eQTL genes among those whose regulation was affected specifically by infection with MTB, the identity of the gene renders the observation intriguing. *CMAS* (cytidine monophosphate N-acetylneuraminic acid synthetase), is an enzyme that is involved in the processing of sialic acid, which is then added to cell surface glycoproteins and glycolipids. Glycoproteins are known to be important in many functions of the immune response, including initial pathogen detection (e.g. TLRs) and antigen presentation (e.g. major histocompatibility complex (MHC) molecules)^104^,^105^,^106^. We suggest that this gene is an interesting candidate for further understanding both MTB pathogenesis and inter-individual susceptibility to tuberculosis.

## Conclusions

By jointly considering data from multiple infection treatments, using a variety of bacteria, we have classified distinct innate immune transcriptional response patterns. The most inclusive pattern was a response to all the bacterial infections, indicating that the receptors that bind the diverse antigens present on the different bacteria converge to largely similar signaling pathways. We also found an expression response pattern specific to mycobacterial infections, the main focus of the current study. At 18 hours post-infection, the mycobacteria response pattern includes genes involved in phagosome maturation and the NAPDH oxidase subunit *NCF2*. At 48 hours post-infection, it includes genes involved in the response to vitamin D and the chemokine *CCL1*. We found that the response to infection with different MTB strains was highly similar. Furthermore, the differences we identified between the response to MTB and the vaccine strain BCG were not mycobacteria-specific, but likely represent a difference between the innate immune response to virulent and non-virulent (or attenuated) pathogens. Lastly, we identified a single gene, *CMAS*, which is both associated with a response eQTL to MTB infection, and whose regulation is altered specifically when we infected the cells with mycobacteria. This gene is thus an especially promising candidate for future studies of TB susceptibility.

## Methods

### Ethics Statement

Buffy coats were obtained from healthy donors after informed consent. The blood collection protocols were approved by both the French Ministry of Research and a French Ethics Committee under the reference DC-2008-68 collection 2. The blood collection was carried out in accordance with these approved protocols by the Etablissement Français du Sang.

### Sample collection and macrophage differentiation

We collected buffy coats (~50 mL) from six healthy donors. Next we isolated peripheral blood mononuclear cells (PBMCs) via Ficoll-Paque centrifugation^38^ and enriched for monocytes via positive selection with beads containing CD14 antibodies^37^. Then we differentiated the monocytes into macrophages by culturing for 6–7 days in RPMI buffer supplemented with macrophage colony-stimulating factor (M-CSF)^107^.

### Bacterial infection

For each bacterial infection (Table 1), we treated the macrophages with a multiplicity of infection (MOI) of 2:1. After one hour, we washed the macrophages five times with phosphate-buffered saline (PBS) and treated them with gentamycin (50 μg/μL) to kill all extracellular bacteria. After one hour of antibiotic treatment, we changed the medium to a lower concentration of gentamycin (5 μg/μL), which marked the zero timepoint of the study. We allowed the cells to grow for 4, 18, or 48 hours before lysing them with QIAzol Lysis Reagent and then storing them at −80 °C. We chose these timepoints based on a previous analysis of the human transcriptional response to infection with MTB^108^. No data is available for 48 hours post-infection with *S. epidermidis*. After escaping the macrophages upon cell death, sufficient *S. epidermidis* were able to proliferate in the gentmycin-supplemented medium to contiminate the entire well by 48 hours post-infection.

### RNA extraction, library preparation, and sequencing

We extracted RNA using the QIAgen miRNeasy kit. There were a total of 13 batches of 12 samples each (6 individuals ×9 conditions ×3 timepoints, minus 48 hours post-infection with *S. epidermidis*). We designed the batches to maximally partition the variables of interest (individual, condition, timepoint) in order to minimize the introduction of biases due to batch processing^109^. To assess RNA quality, we measured the RNA Integrity Number (RIN) with the Agilent Bioanalyzer (Supplementary Table S6). Importantly, there were no significant differences in the RIN (mean of 7.8 ± 2.0) between the bacterial infections or between the timepoints (Supplementary Fig. S9B). In batches of 12 samples, we added barcoded adapters (Illumina TruSeq RNA Sample Preparation Kit v2) and sequenced 50 base pairs single end over multiple flow cells on the Illumina HiSeq 2500.

### Mapping, counting, and normalization

We mapped the short reads to the human genome (hg19) using the Subread algorithm^40^ and discarded those that mapped non-uniquely. Next, we obtained the read counts for each Ensembl protein-coding gene (biotype: “protein_coding”) with the featureCounts algorithm, which sums the reads falling in the union of all exons of a gene and discards reads mapping to more than one gene^110^. There were no significant differences in the number of mapped exonic reads (mean of 41.8 ± 21.2 million per sample) between the bacterial infections or between the timepoints (Supplementary Fig. S9A). We removed genes with fewer than one count per million exonic reads in fewer than six samples. To account for differences in the read counts at the extremes of the distribution, we normalized the samples using the weighted trimmed mean of M-values algorithm (TMM)^41^.

### Differential expression analysis

To assess the quality of the data, we performed principal components analysis (PCA) of the TMM-normalized log_2_-transformed counts per million (CPM). PC2 separated the samples by timepoint, but PC1 was associated with the RIN score and the processing batch (Supplementary Fig. S2A). After the effects of RIN score and processing batch were removed with the function removeBatchEffect from the limma package^111^, PC1 separated the samples by timepoint and PC2 separated the infected and control samples (Supplementary Fig. S2B). We protected the variables of interest (individual, bacteria, timepoint) when regressing the effects of RIN score and processing batch by including them in the linear model used by removeBatchEffect. However, the result was similar if they were not protected since the variables of interest were partitioned across the processing batches (Supplementary Fig. S2C). All figures displaying expression data were generated using the batch-corrected data.

To confirm that the transcriptional response to MTB infection in our study was consistent with previous observations, we compared our MTB infected samples and their time-matched controls to the MTB infected samples and zero timepoint control from Tailleux *et al.*, 2008^108^. Despite differences in the technology used to assay gene expression (RNA-seq versus microarray) and the method used to isolate the macrophages (positive versus negative selection), we still observed a common transcriptional signature of infection using PCA (Supplementary Fig. S10).

For the standard analysis, we tested for differential expression using limma + voom^42^,^43^,^44^ because it has been shown to perform well with sufficient sample size (n > = 3 per condition)^112^,^113^. Based on the PCA results, we included RIN score and processing batch as covariates in the model. We corrected for multiple testing with the Benjamini & Hochberg false discovery rate (FDR)^114^ and considered genes with q-value less than 5% to be differentially expressed.

Since we were interested in the shared and differential response to infection with the different bacteria, we performed a joint Bayesian analysis using the Cormotif algorithm^45^. Cormotif shares information across experiments, in this case infections, to identify the main patterns of differential gene expression (which it refers to as *correlation motifs*) and assigns each gene to one of these gene expression patterns. One caveat of the Cormotif algorithm is that is does not distinguish the direction of the effect across infections. In other words, a gene that is assigned to an expression pattern could be differentially expressed in different directions across the infections. However, in this data set, this was rarely observed (Supplementary Table S9).

In practice, we had to make several modifications when using Cormotif. First, since the method was developed for microarray data, we used the batch-corrected TMM-normalized log_2_-transformed CPM as input. Second, the method assumes independence between the experiments, and we only have one control per timepoint. However, since this dependence will cause genes to be more likely to be either uniformly differentially expressed across all the infections or uniformly unchanged, this caveat is conservative to our results of gene expression patterns that are specific to subgroups of the bacterial infections. Third, the current version of the method (v1.14.0) does not return the cluster likelihoods, i.e. the likelihood that a gene belongs to each of the gene expression patterns. To facilitate downstream analyses with these sets of genes, we modified the original code to additionally return this information. Lastly, Cormotif is non-deterministic. Thus to obtain consistent results, we ran each test 100 times and kept the result with the largest maximum likelihood estimate.

We tested for enrichment of gene ontology (GO) biological processes among the genes in the gene expression patterns using topGO^115^. We tested for significance with the Fisher's Exact Test, used the weight01 algorithm from topGO to account for the correlation among GO categories due to its graph structure, and considered significant any category with p-value less than 0.01.

### Analysis using previously identified response eQTLs

We downloaded the list of response eQTL genes from Supplementary Table 3 from Barreiro *et al.*^37^. Of the 198 response eQTL genes discovered in the dendritic cells in that study, 179 of the genes were also expressed in the macrophages from this study. In order to compare the differential expression results of the response eQTL genes to other genes, we used the log_2_ fold changes in expression estimated by limma^111^. First, we calculated the mean log_2_ fold change at 18 hours post-infection for each gene across the eight bacteria. Second, we converted these mean estimates to their absolute values. Third, we subsampled 100 genes from each of the three categories (response eQTL, general eQTL, and non-eQTL genes) and calculated the mean of the absolute values. We performed this subsampling 1000 times (Fig. 5B). Fourth, we performed t-tests to compare the distribution of response eQTL genes to either that of the general eQTL genes or the non-eQTL genes.

### Data and code availability

The data have been deposited in NCBI's Gene Expression Omnibus^116^ and are accessible through GEO Series accession number GSE67427 (http://www.ncbi.nlm.nih.gov/geo/query/acc.cgi?acc=GSE67427). Supplementary Table S1, which contains the gene expression data, and Supplementary Table S2, which contains the differential expression results from limma, are available from our lab website: http://giladlab.uchicago.edu. The code is available at https://bitbucket.org/jdblischak/tb.

## Additional Information

**How to cite this article**: Blischak, J. D. *et al.* Mycobacterial infection induces a specific human innate immune response. *Sci. Rep.* **5**, 16882; doi: 10.1038/srep16882 (2015).

## Supplementary Material

Supplementary Information

Supplementary Table S3

Supplementary Table S4

Supplementary Table S5

Supplementary Table S6

Supplementary Table S7

Supplementary Table S8

Supplementary Table S9

## Figures and Tables

**Table 1:** Description of bacteria.

Abbr.	Name	Description	Gram staining*
none	control	Mock infection	N/A
Rv	MTB H37Rv	A common laboratory strain of MTB	acid-fast
Rv+	heat-inactivated MTB H37Rv	Dead MTB H37Rv	acid-fast
GC	MTB GC1237	More virulent strain of MTB	acid-fast
BCG	bacillus Calmette-Guérin	Vaccine (attenuated *M. bovis*)	acid-fast
Smeg	*Mycobacterium smegmatis*	Non-pathogenic mycobacterium	acid-fast
Yers	*Yersinia pseudotuberculosis*	Facultative intracellular pathogen	Negative
Salm	*Salmonella typhimurium*	Facultative intracellular pathogen	Negative
Staph	*Staphylococcus epidermidis*	Extracellular pathogen	Positive

^*^Mycobacteria are unable to be gram stained due to the low permeability of their cell walls. They are more closely related evolutionarily to gram-positive bacteria than gram-negative. However, their thick cells walls share features of gram-negative bacteria, e.g. a "pseudoperiplasm" similar to the gram-negative periplasm^117^.
