# Supplementary material for: Mycobacterial infection induces a specific human innate immune response
Source: Sci Rep. 2015 Nov 20;5:16882. doi: 10.1038/srep16882 (PMC4653619; doi:10.1038/srep16882)
Supplement: Supplementary Information [file srep16882-s1.pdf]

Supplementary Information

Mycobacterial infection induces a specific human innate immune response

John D. Blischak[1,2], Ludovic Tailleux[3], Amy Mitrano[1], Luis B. Barreiro[4,5]*, Yoav Gilad[1]*

[1]Department of Human Genetics, University of Chicago, Chicago, Illinois, USA

[2]Committee on Genetics, Genomics, and Systems Biology, University of Chicago, Chicago, Illinois, USA

[3]Mycobacterial Genetics Unit, Institut Pasteur, Paris, France

[4]Department of Genetics, CHU Sainte-Justine Research Center, Montreal, Québec, Canada

[5]Department of Pediatrics, University of Montreal, Montreal, Québec, Canada

*Correspondence should be addressed to YG (gilad@uchicago.edu) and LBB (luis.barreiro@umontreal.ca).

Supplementary Tables

Supplementary Table S1 - available for download from our lab website

Supplementary Table S1 contains the batch-corrected $\log_2$ counts per million for the 12,728 Ensembl genes analyzed in this study for each of the 156 samples. The column names are in the format "individual.infection.time". It can be downloaded from http://giladlab.uchicago.edu or https://bitbucket.org/jdblischak/tb-data.

(TXT)

Supplementary Table S2 - available for download from our lab website

Supplementary Table S2 contains the differential expression statistics from limma. This includes the $\log_2$ fold change (logFC), average expression level (AveExpr), t-statistic (t), p-value (P.Value), q-value (adj.P.Val), and log-odds (B). The column names also contain the infection and timepoint for the given comparison. It can be downloaded from http://giladlab.uchicago.edu or https://bitbucket.org/jdblischak/tb-data.

(TXT)

Supplementary Table S3 - provided as a separate file

Supplementary Table S3 contains the assigned expression patterns for the 12,728 Ensembl genes analyzed in this study for each of the three analyses in Figures 2, 3, and 4. The columns "full_time_course", "time_18h", and "time_48h" correspond to Figures 2, 3, and 4, respectively.

(TDS)

Supplementary Table S4 - provided as a separate file

Supplementary Table S4 contains the assigned expression patterns for the 12,728 Ensembl genes analyzed in this study for each of the three analyses in Figures 2, 3, and 4. It is the same information as Supplementary Table S3, but with the genes from each pattern from each of the three Figures in its own sheet of the workbook. Furthermore, it contains the gene descriptions from Ensembl.

(XLS)

Supplementary Table S5 - provided as a separate file

Supplementary Table S5 contains the gene ontology results for each of the expression patterns for the three analyses in Figures 2, 3, and 4.

(XLS)

Supplementary Table S6 - provided as a separate file

Supplementary Table S6 contains the RNA Integrity Number (RIN) and molarity (nmol/L) measured with a Bioanalyzer (Agilent) for each of the 156 samples.

(TDS)

Supplementary Table S7 - provided as a separate file

Supplementary Table S7 contains the results of intersecting lists of differentially expressed genes for all pairwise comparisons (within each of the three timepoints).

(TDS)

Supplementary Table S8 - provided as a separate file

The number of differentially expressed genes when performing all pairwise tests between bacterial infections for each of the three timepoints.

(XLS)

Supplementary Table S9 - provided as a separate file

Cormotif does not distinguish between the direction of the effect when assigning a gene to a given expression pattern. For example, a gene that is upregulated in one infection but downregulated in another is indistinguishable from a gene that is upregulated in response to both infections. However, in this data set, this is a rare effect. We calculated the percent concordance for the genes in the expression patterns from the three separate analyses. For example, for the expression pattern "MTB", 100% would indicate the gene is regulated in the same direction in the five mycobacterial infections, 80% would indicate that the gene is regulated in the same direction for four of the five mycobacterial infections, etc. "num_concord" is the number of genes in that expression pattern that are 100% concordant across the infections. "num_discord" is the number of genes in that expression pattern that are not 100% concordant. "mean_perc_concord" is the mean percent concordance of all the genes in that expression pattern.

(TDS)

Supplementary Figures

Supplementary Figure S1

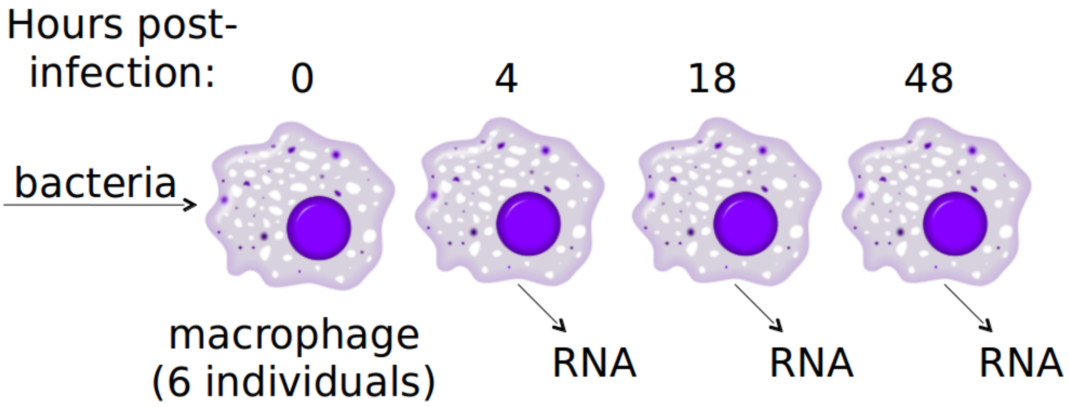

Supplementary Figure S1. Study design.

We infected monocyte-derived macrophages isolated from six healthy donors with the bacteria described in Table 1. We isolated RNA for sequencing at 4, 18, and 48 hours post-infection.

Supplementary Figure S2

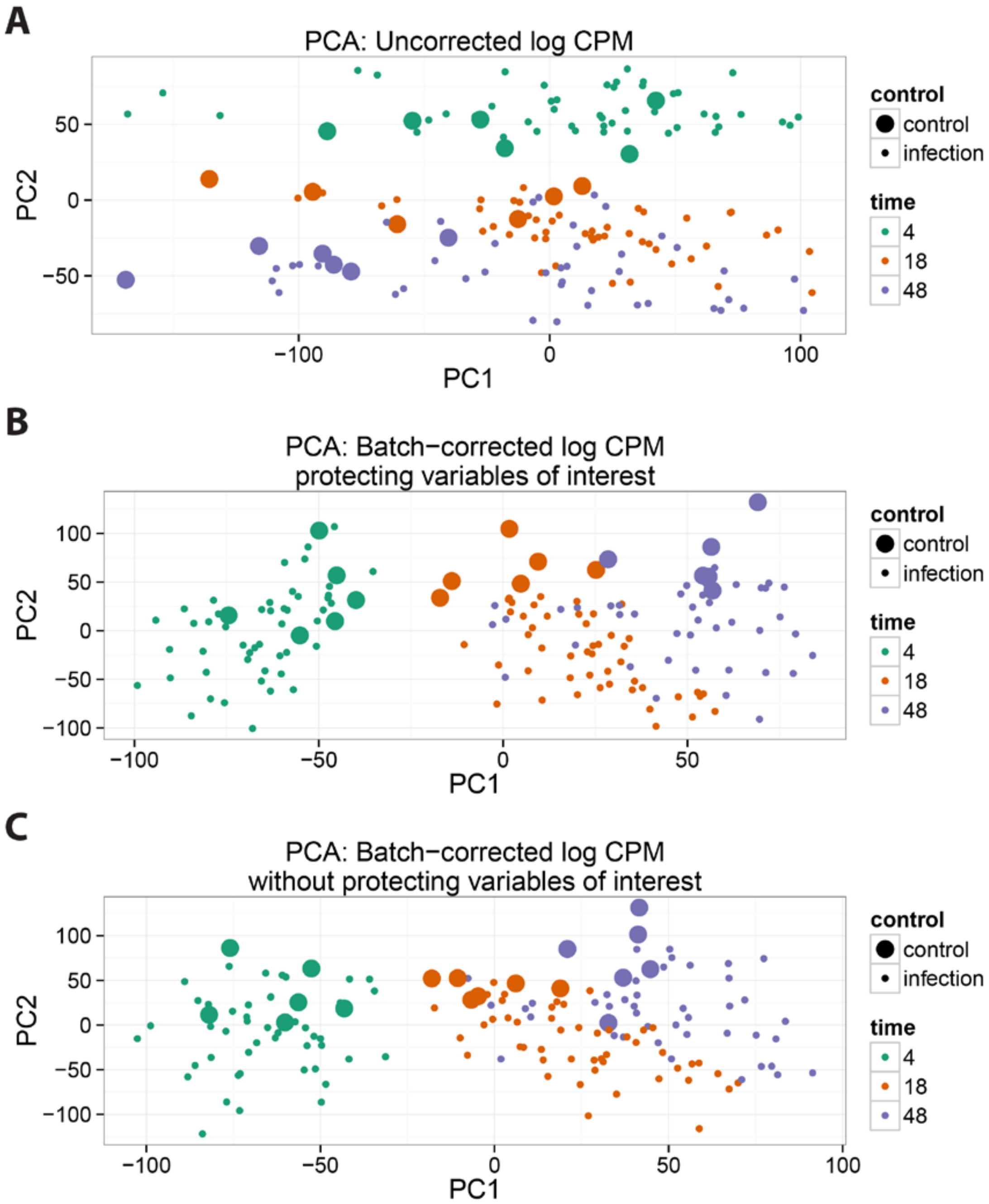

Supplementary Figure S2. Principal components analysis (PCA) of uncorrected and batch-corrected expression values.

A) PCA of the TMM-normalized log_2-transformed counts per million (CPM). Infected and control samples are not well separated. PC2 separates the samples by timepoint.

B) PCA of the TMM-normalized log_2-transformed CPM after removing the effects of RIN score and processing batch. PC1 separates the samples by timepoint. PC2 separates the infected and control samples.

C) PCA of the TMM-normalized log_2-transformed CPM after removing the effects of RIN score and processing batch without protecting the variables of interest (individual, bacteria, timepoint).

Supplementary Figure S3

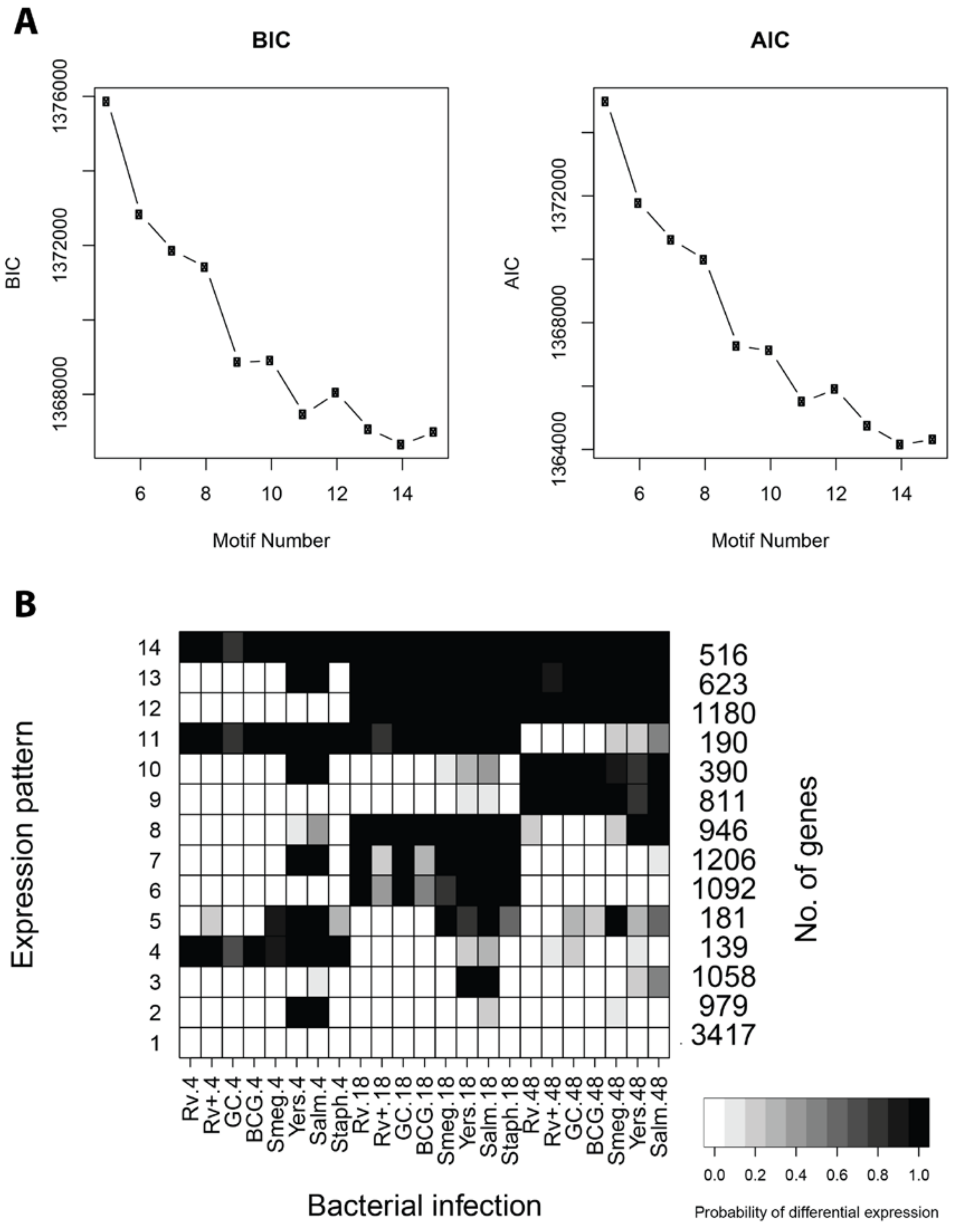

Supplementary Figure S3. Joint Bayesian analysis with 14 expression patterns.

(A) Cormotif[1] estimates the number of expression patterns (i.e. motifs, using their terminology) to use by calculating the Bayesian information criterion (BIC) and the Akaike information criterion (AIC). These criteria penalize models for additional parameters to avoid overfitting. The model with the lowest BIC/AIC is considered the best fit, which in this context is the model with 14 expression patterns.

(B) Joint analysis with Cormotif. The shading of each box represents the posterior probability that a gene assigned to the expression pattern (row) is differentially expressed in response to infection with a particular bacteria (column), with black representing a high posterior probability and white a low posterior probability. The expression patterns have the following interpretations:

1. "non-DE" - Genes that do not respond to infection.
2. "Yers-Salm-4h" - Genes that respond 4 hours post-infection with *Y. pseudotuberculosis* or *S. typhimurium*.
3. "Yers-Salm-18h" - Genes that respond 18 hours post-infection with *Y. pseudotuberculosis* or *S. typhimurium*.
4. "4h" - Genes that respond to 4 hours post-infection with any bacteria.
5. "non-MTB" - Genes that respond at 4, 18, and 48 hours post-infection to bacteria that are not MTB or BCG (attenuated *M. bovis*).
6. "Virulent-18h" - Genes that respond 18 hours post-infection with virulent bacteria.
7. "Virulent-18h+Yers-Salm-4h" - Genes that respond 18 hours post-infection with virulent bacteria and 4 hours post-infection with *Y. pseudotuberculosis* or *S. typhimurium*.

8. "18h+Yers-Salm-48h" - Genes that respond 18 hours post-infection with any bacteria and 48 hours post-infection with *Y. pseudotuberculosis* or *S. typhimurium*.
9. "48h" - Genes that respond 48 hours post-infection with any bacteria.
10. "48h+Yers-Salm-4h" - Genes that respond 48 hours post-infection with any bacteria and 4 hours post-infection with *Y. pseudotuberculosis* or *S. typhimurium*.
11. "4&18h" - Genes that respond 4 and 18 hours post-infection with any bacteria.
12. "18&48h" - Genes that respond 18 and 48 hours post-infection with any bacteria.
13. "18&48h+Yers-Salm-4h" - Genes that respond 18 and 48 hours post-infection with any bacteria and 4 hours post-infection with *Y. pseudotuberculosis* or *S. typhimurium*.
14. "All" - Genes that respond at 4, 18, and 48 hours post-infection with any bacteria.

Supplementary Figure S4

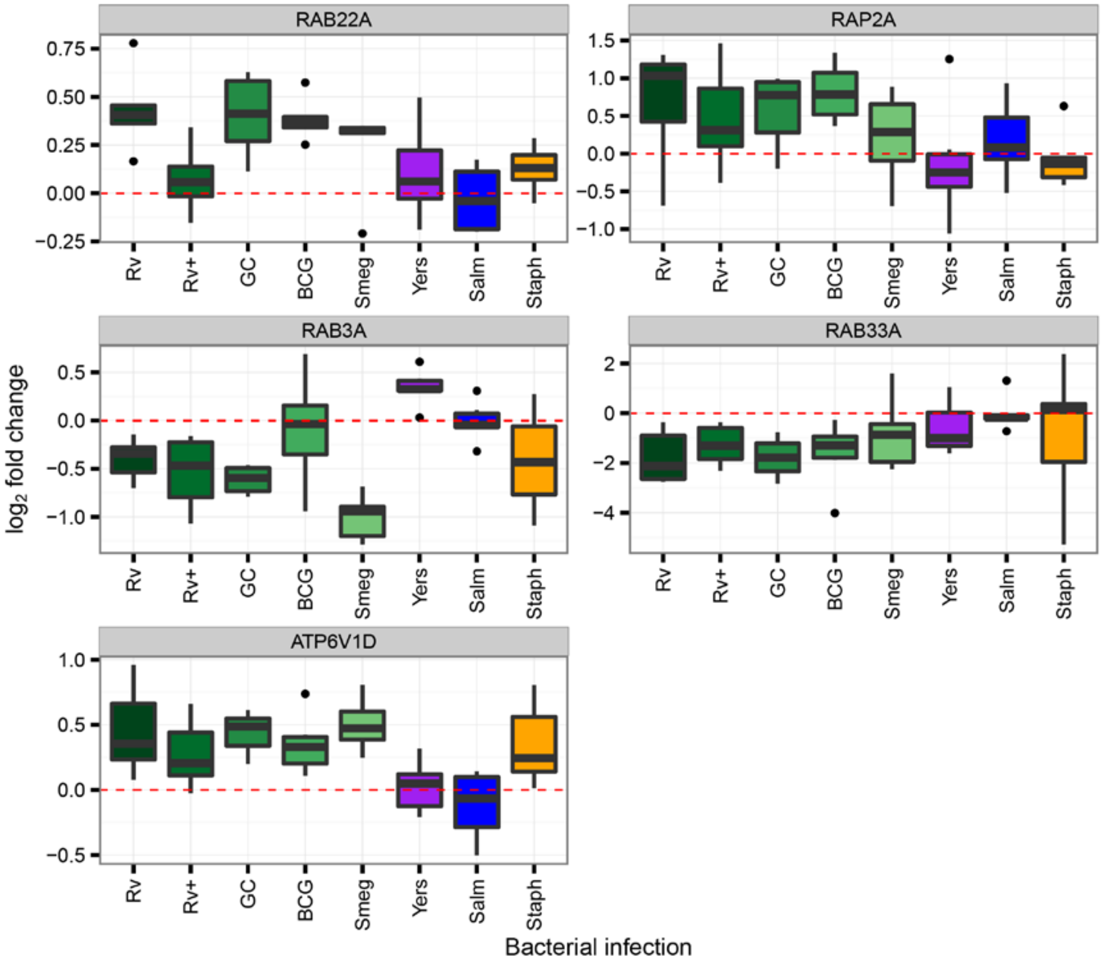

Supplementary Figure S4. Expression of genes involved in phagosome maturation.

RAB22A, *RAP2A*, and *ATP6V1D* are upregulated in response to infection with mycobacteria at 18 hours; whereas, *RAB3A* and *RAB33A* are downregulated (pattern "MTB" in Figure 3).

Supplementary Figure S5

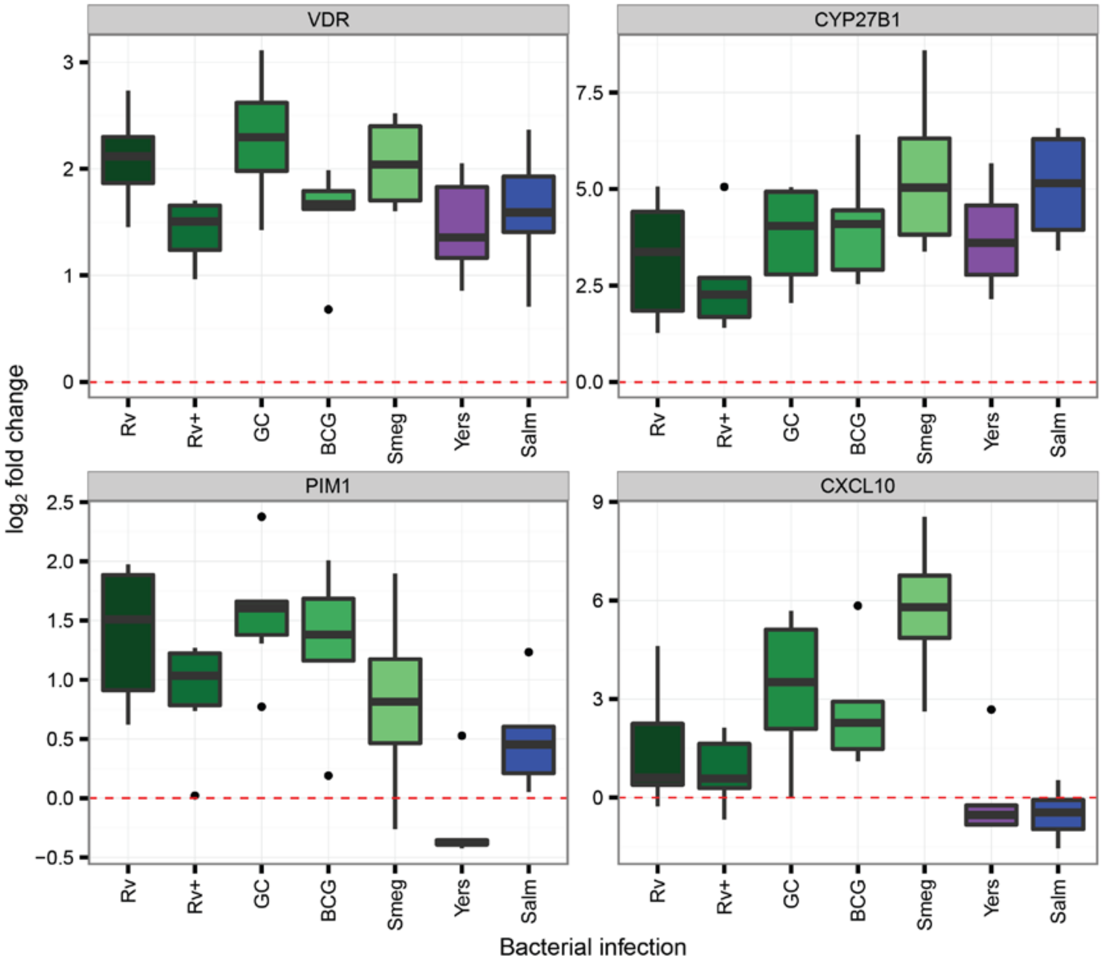

Supplementary Figure S5. Expression of genes involved in vitamin D signaling.

VDR and *CYP27B1* are upregulated at 48 hours post-infection with all bacteria (pattern "All" in Figure 4). *PIM1* and *CXCL10* are upregulated at 48 hours post-infection with the mycobacteria (pattern "MTB" in Figure 4).

Supplementary Figure S6

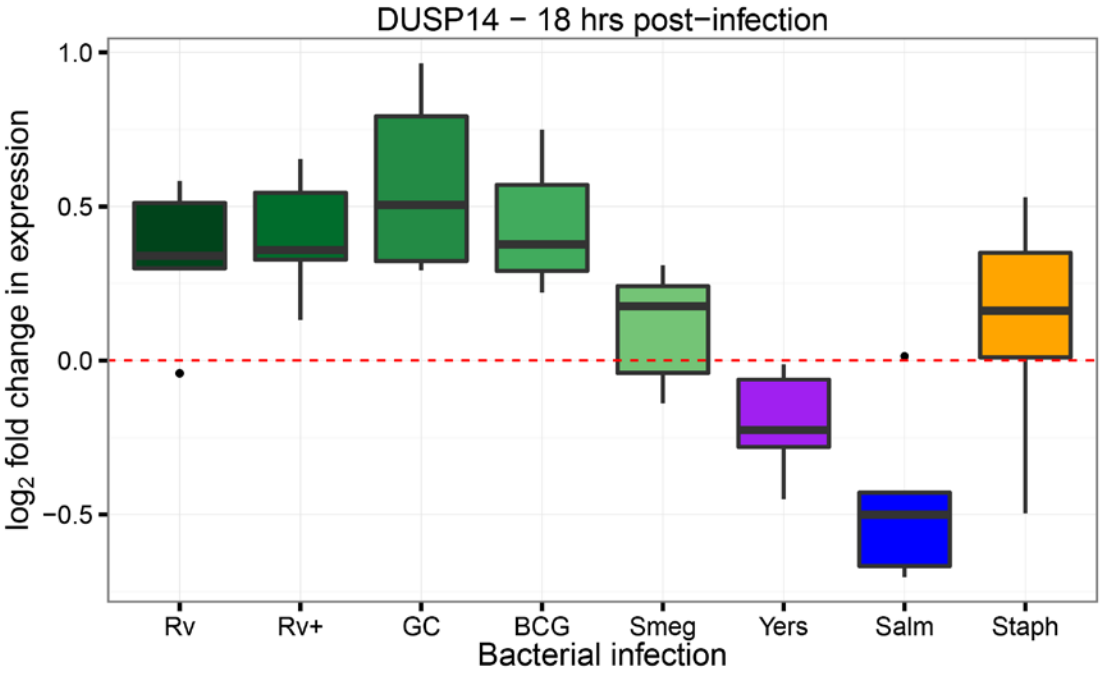

Supplementary Figure S6. Expression of *DUSP14* at 18 hours post-infection.

DUSP14 is an example of an interesting gene not identified with our approach. At 18 hours, it is upregulated after infection with MTB H37Rv (q-value: 16%), MTB GC1237 (q-value: 3%), and BCG (q-value: 9%) (the change in heat-inactivated MTB H37Rv had a q-value of 26%); and downregulated post-infection with *S. typhimurium* (q-value: 9%). Because it did not fit well into one of the main patterns of gene expression identified at 18 hours post-infection, it was classified as the "non-DE" pattern.

Supplementary Figure S7

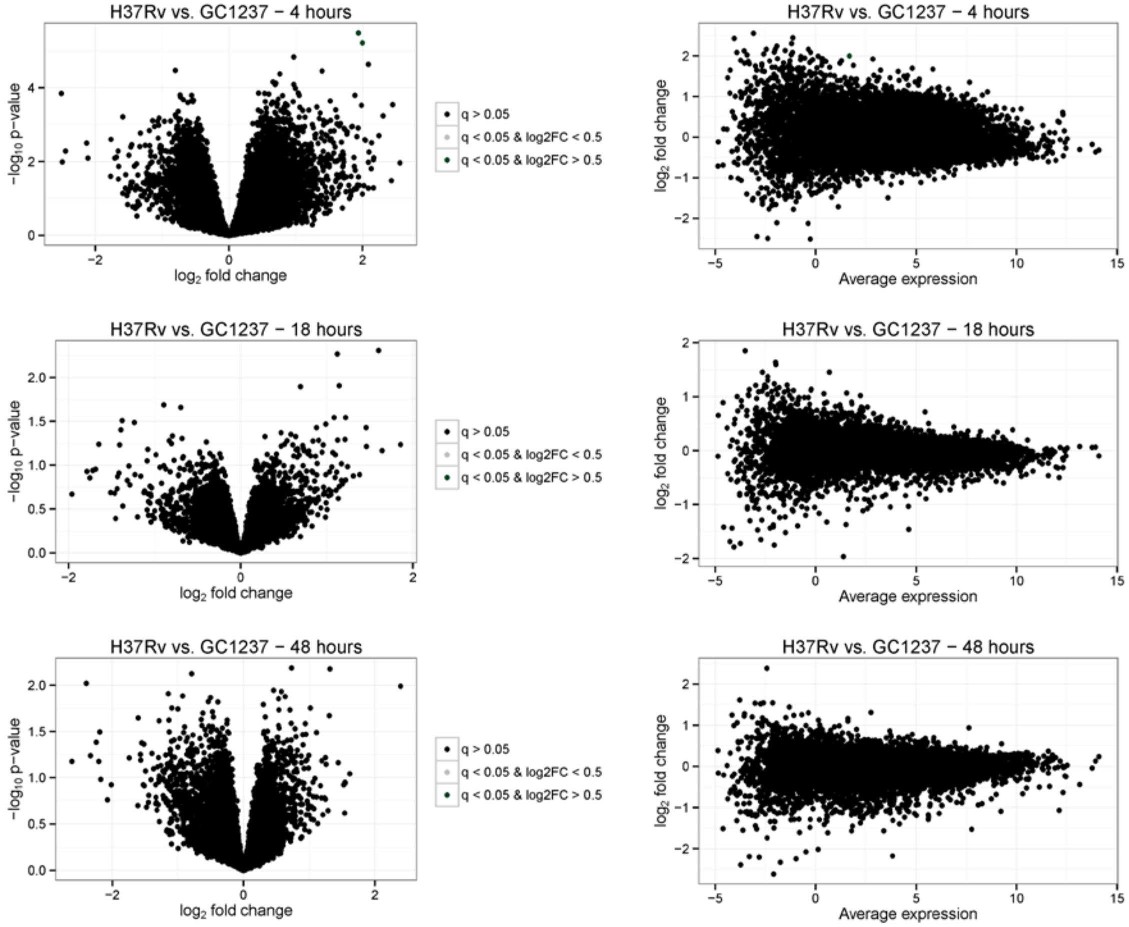

Supplementary Figure S7. Little difference in transcriptional response to infections with different MTB strains.

Few statistically significant differences were identified when explicitly testing gene expression levels post-infection with MTB H37Rv and MTB GC1237 at 4, 18, or 48 hours post-infection (top, middle, and bottom panels, respectively). The volcano plots (left) display the -$\log_{10}$ transformed p-value versus the $\log_2$ fold change in expression level. The MA plots (right) display the $\log_2$ fold change in expression level versus the average expression level. Most of the genes are labeled black indicating that their FDR value is greater than 5%. Two genes (labeled

green) at 4 hours post-infection had a q-value < 0.05 and $\log_2$ fold change greater than 0.5 (see Supplementary Table S8).

Supplementary Figure S8

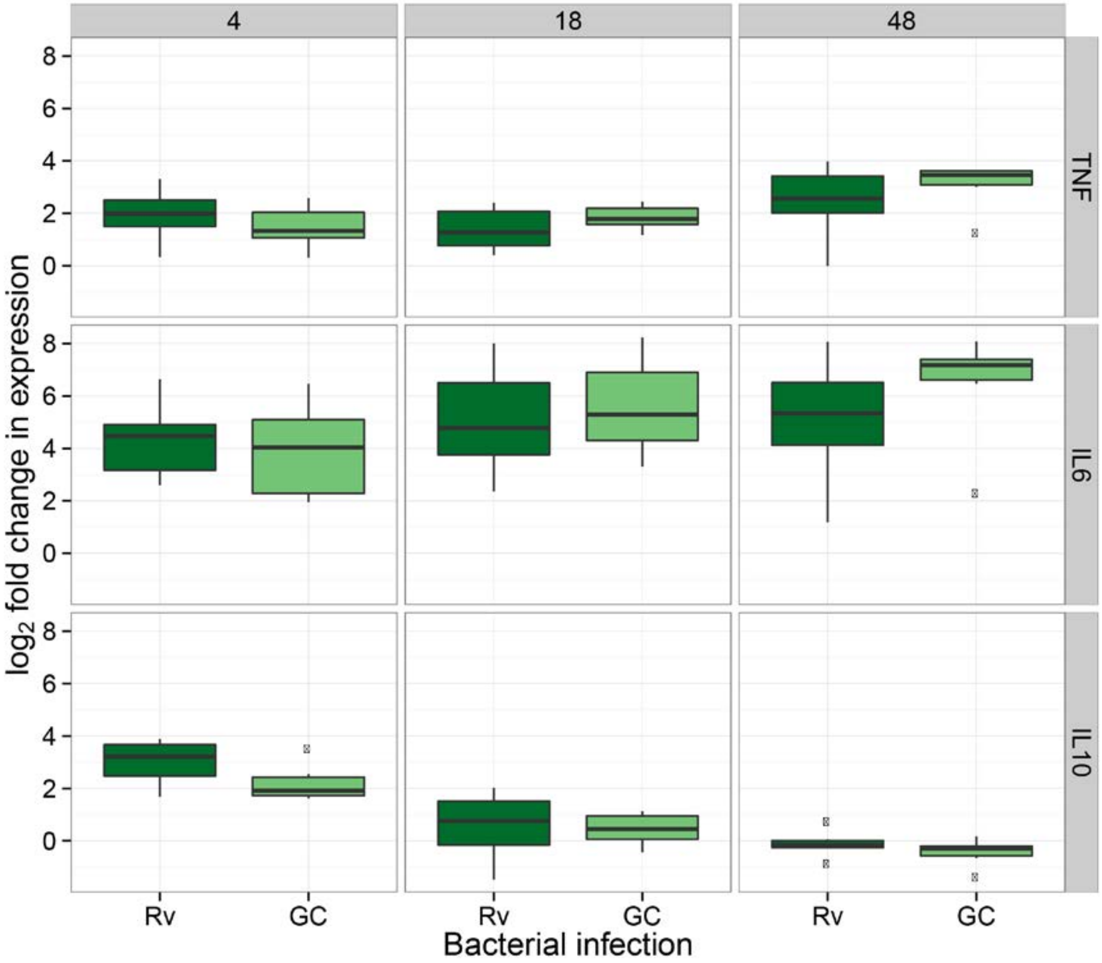

Supplementary Figure S8. Response of example cytokines to infection with different MTB strains.

The $\log_2$ fold change in expression of the pro-inflammatory cytokines *TNF* and *IL6* and the anti-inflammatory cytokine *IL10* is similar post-infection with MTB H37Rv or MTB GC1237. *TNF* and *IL6* are upregulated at all three timepoints; whereas, *IL10* is upregulated only at 4 hours post-infection.

Supplementary Figure S9

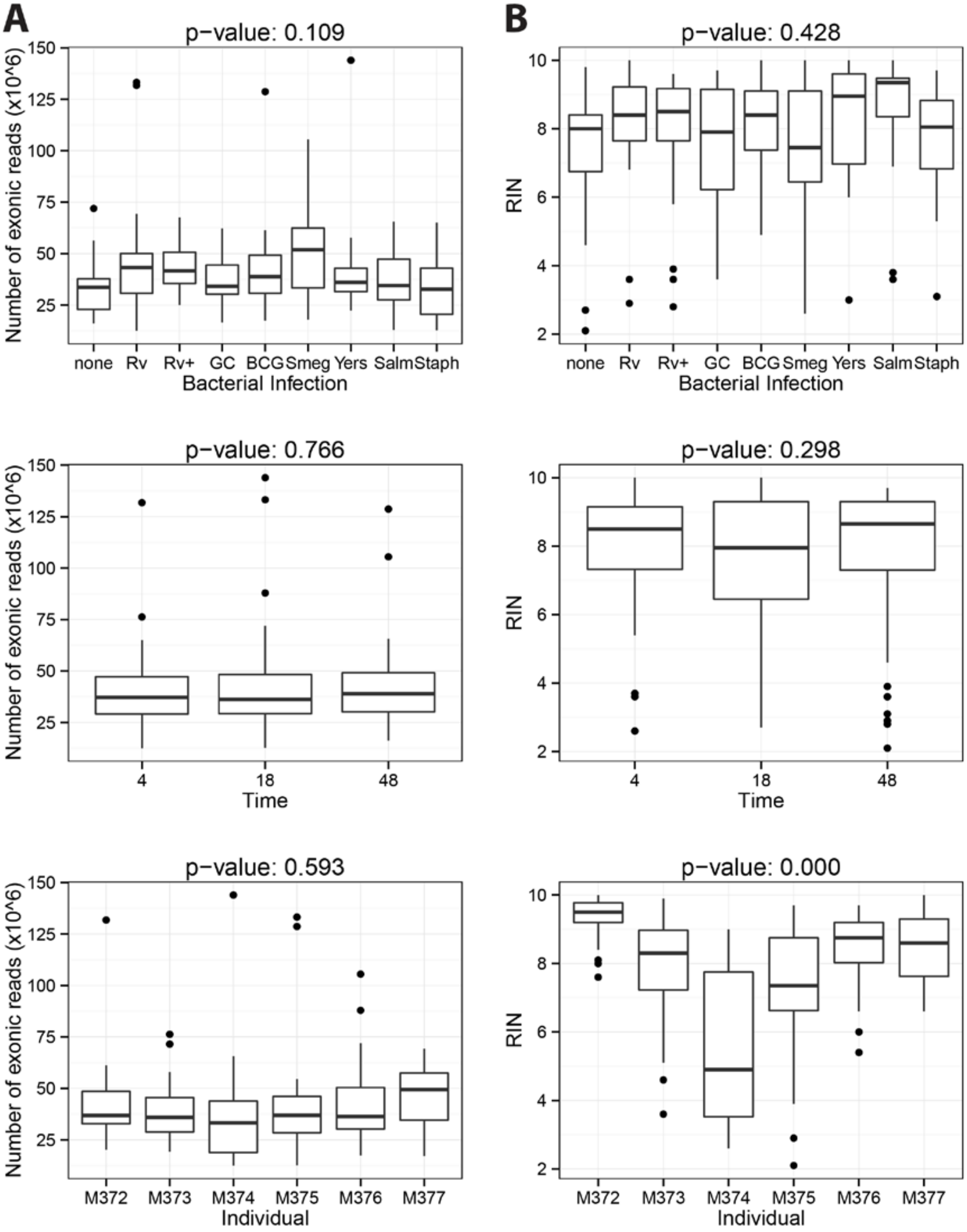

Supplementary Figure S9. Distribution of the number of exonic reads and RNA quality scores (RIN) across variables of interest.

A) The number of exonic reads is evenly distributed across the bacterial infections, timepoints, and individuals.

B) The RIN scores are evenly distributed across the bacterial infections and timepoints; however, the RIN does vary between the individuals. The p-values are from an F-test.

Supplementary Figure S10

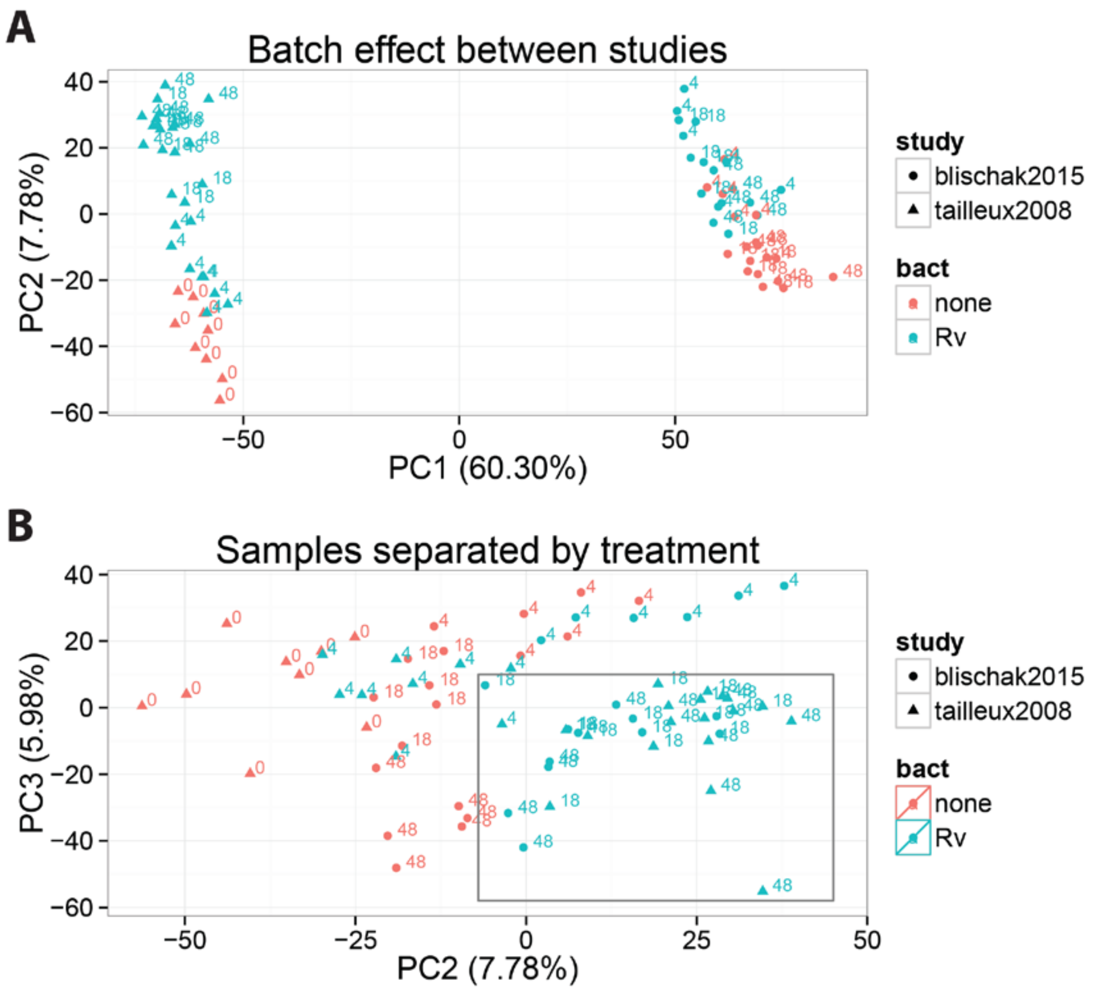

Supplementary Figure S10. Comparison to Tailleux et al., 2008.

We compared our RNA-seq data to the microarray data of Tailleux et al., 2008[2] to confirm a consistent signature of infection. From our experiment, we used the batch-corrected TMM-normalized log_2-transformed counts per million (CPM) from the MTB H37Rv infected macrophages at 4, 18, and 48 hours post-infection and their time-matched controls. From their experiment, we used the log_2-transformed quantile-normalized data from the MTB H37Rv infected macrophages at 4, 18, and 48 hours post-infection as well as the zero timepoint non-infection control. In addition to the difference in technology, the macrophages were isolated via positive selection in our study and negative selection in theirs. Despite these differences, we still observe a common transcriptional signature of infection when performing principal components analysis (PCA).

A) PC1 is the expected batch effect between the two experiments.

B) Plotting PC2 versus PC3, the infected samples at 18 and 48 hours (when there is a strong transcriptional response; see Figure 1A) from the two different studies cluster together.

The quantile-normalized data from Tailleux et al., 2008[2] is available at https://bitbucket.org/jdblischak/tb-data.

References

1.Wei, Y., Tenzen, T. & Ji, H. Joint analysis of differential gene expression in multiple studies using correlation motifs. *Biostatistics (Oxford, England)* **16,** 31–46 (2015).

2.Tailleux, L. *et al.* Probing host pathogen cross-talk by transcriptional profiling of both Mycobacterium tuberculosis and infected human dendritic cells and macrophages. *PloS one* **3,** e1403 (2008).
